# Ambipolar inverters based on cofacial vertical organic electrochemical transistor pairs for biosignal amplification

**DOI:** 10.1126/sciadv.abh1055

**Published:** 2021-09-08

**Authors:** Reem B. Rashid, Weiyuan Du, Sophie Griggs, Iuliana P. Maria, Iain McCulloch, Jonathan Rivnay

**Affiliations:** 1 Department of Biomedical Engineering, Northwestern University, Evanston, IL 60208, USA.; 2 Simpson Querrey Institute, Northwestern University, Chicago, IL 60611, USA.; 3 Physical Sciences and Engineering Division, KAUST Solar Center (KSC), King Abdullah University of Science and Technology (KAUST), Thuwal 23955-6900, Saudi Arabia.; 4 Department of Chemistry, Chemistry Research Laboratory, University of Oxford, Oxford OX1 3TA, UK.

## Abstract

On-site signal amplification for bioelectronic sensing is a desirable approach to improving recorded signal quality and to reducing the burden on signal transmission and back-end electronics. While organic electrochemical transistors (OECTs) have been used as local transducers of bioelectronic signals, their current output presents challenges for implementation. OECT-based circuits offer new opportunities for high-performance signal processing. In this work, we introduce an active sensing node based on cofacial vertical OECTs forming an ambipolar complementary inverter. The inverter, which shows a voltage gain of 28, is composed of two OECTs on opposite side walls of a single active area, resulting in a footprint identical to a planar OECT. The inverter is used as an analog voltage preamplifier for recording electrocardiogram signals when biased at the input voltage corresponding to peak gain. We further demonstrate compatibility with nontraditional fabrication methods with potential benefits for rapid prototyping and large-area printed electronics.

## Teaser

A compact circuit based on electrochemical transistors for measuring and amplifying electrophysiological signals.

## INTRODUCTION

Bioelectronic recordings traditionally decouple front-end signal transduction (biochemical, biophysical, and electrophysiological detection) from downstream signal processing (amplification, filtering, or feature detection) (*1*, *2*). Minimizing the physical distance and improving system integration between components responsible for signal transduction and processing can improve recorded signal quality, simplify implementation, and, more readily, enable practical wireless systems (*3*, *4*). The typical approach for on-board signal processing requires silicon complementary metal-oxide semiconductor (CMOS) circuits in the form of packaged or bare die silicon integrated circuits or, alternatively, front-end colocalization of sensors and CMOS circuits (*3*, *5*–*9*). Imparting additional functionality to a sensing node is an approach gaining notable attention, as it may lead to yet more integrated, higher performing, and lower power systems (*1*, *4*, *6*). Additional functionality can include multimodal or multimarker sensors that allow for measurement of simultaneous complementary biomarkers at the same sensing site or on-site analog signal processing (*4*, *10*–*12*). This on-site or in-sensor signal processing could further provide instantaneous feedback and enable future low power closed-loop devices that perform diagnostic and treatment functions (*13*, *14*). Achieving active sensing nodes often requires trade-offs between performance/functionality and physical footprint, which can, for example, yield lower density arrays, and requires fabrication and assembly approaches not easily achievable with flexible or conformable form factors (*1*, *6*, *15*). To realize compact form factors and amplification at a single sensing site, we turn to recent developments in organic electrochemical transistors (OECTs), a rising class of polymer-based bioelectronic components.

OECTs are transistors based on organic mixed conductors that have gained attention in the field of bioelectronics due to their role as efficient ionic-to-electronic transducers (*16*, *17*). OECTs have been used in a variety of biological applications, ranging from neural recordings to metabolite sensing, while outperforming inorganic transistor and electrode-based sensors (*17*–*19*). As with other transistors, OECTs are three terminal devices (drain, source, and gate terminals) with a typically polymeric channel between the source and drain (S/D) (*16*, *20*). The bias on the gate terminal induces ionic drift into the semiconducting polymer channel, effectively dedoping/doping the film, while the current collected at the drain terminal probes this modulation (*16*). Small changes in the gate bias result in large changes in the drain current, leading to high transconductance (*g*_m_), which translates to high local voltage to current amplification (*16*, *17*, *21*). Bulk mixed conduction leads to larger amplification as compared to transistors, which rely on a double-layer charging for devices of comparable size (*16*, *19*, *22*). Furthermore, improved mechanical matching with biological tissue, operation in aqueous environments, and ease of fabrication make OECTs attractive for biological applications (*16*). The bulk transport of OECTs eases fabrication requirements, allowing for high performance using fabrication techniques such as inkjet printing and laser cutting, resulting in unique form factors and device concepts (*22*–*26*). In addition, the low operational voltage of OECTs leads to lower power consumption and lower heat dissipation, which are important for system integration and safety (*16*, *27*).

Despite recent advances in materials understanding, device physics, and single OECT bioelectronic implementation, OECT integration into simple circuits is needed to achieve desired functionality. OECT signal processing circuits have been pursued for the purposes of amplification to voltage thresholding (*4*, *17*, *19*, *28*, *29*). One of the first OECT-based analog circuits was a voltage amplifier, consisting of an OECT and resistor in series, to convert the current output of the OECT into a voltage (*30*, *31*). Current is not typically compatible with downstream electrophysiology equipment and data analysis software (*30*). In addition, the voltage amplifier can be used to drive subsequent OECTs in cascaded amplifiers for further amplification (*32*). These voltage amplifiers have even been used as the branches in a Wheatstone bridge for reference-based sensing (*28*, *29*). While these approaches using a load resistor to provide a voltage-to-voltage amplification show promise, they generally show higher power dissipation, and the added resistor can contribute additional thermal noise, drawing attention to the use of inverter logic gates as voltage amplifiers.

Digital circuits and logic gates can serve analog signal processing needs as well: Inverters, for example, are also an attractive building block for voltage amplification. An inverter is a logic gate that inverts the input signal applied (low-voltage input leads to a high-voltage output and vice versa) and demonstrates a high gain in the transition region. Their use is more typical for digital logic applications where decoders and multiplexers can be used to ease data handling and reduce overall device area but can be useful for voltage amplification when operated in the transition region (*24*, *26*, *33*). Despite the early use of Poly(3,4-ethylenedioxythiophene) polystyrene sulfonate (PEDOT:PSS) OECTs for (unipolar) resistive ladder-based inverters for numerous digital circuits (*24*, *26*, *33*–*35*), inverters are typically made of a pair of transistors with opposing charge transport (n- and p-type), also known as complementary inverters. Ideal complementary inverters can be more desirable, as they offer lower power consumption and, for digital logic, improved noise margins (*36*). OECT-based complementary inverters have been previously realized by Sun and coworkers (*36*) using a poly-(3-carboxy-pentyl-thiphene) (P3CPT) p-type OECT and a poly(benzimidazobenzophenanthroline) (BBL) n-type OECT. The n- and p-type OECTs were on separate substrates to allow for different channel thicknesses to account for the imbalance in electronic properties between the two OECT materials (*36*). By doing so, Sun *et al*. were able to achieve a balanced inverter with a peak gain of 12; however, the dimensions of the OECTs were on the millimeter scale. Higher gains were achieved by inverters composed of a PEDOT:PSS p-type OECT and a BBL n-type OECT (*37*). These inverters demonstrated a peak gain of about 60; however, the width of the n-type OECT was still on the millimeter scale (*37*). The challenges for the few cases of OECT complementary inverters are the need to pattern multiple active materials, and the physical real estate taken by the transistors needed to achieve on-site amplification.

Vertical OECTs (vOECTs) present a compelling route to decrease transistor footprint and increase recording density. In vOECTs, the S/D contacts are stacked on top of each other vertically rather than laterally on a planar two-dimensional surface (pOECT) (*38*). The interest in vOECTs is not only due to their smaller device footprints but also due to their enhanced electronic properties (*20*, *38*). Higher transconductance and cutoff frequency can be attained with vOECTs with the same or smaller area than a pOECT (*16*, *20*, *22*, *38*). The length of a vOECT channel is nominally defined by the thickness of an insulating interlayer between the S/D contacts for vOECTs, while it is the planar distance between the two for pOECTs, typically defined as photolithographically for microscale OECTs (*38*). The thickness of the insulation can be reliably made submicron while still pinhole free, while the pOECTs are limited by photolithography capabilities and alignment requirements when fabricating via standard photolithography. The first account of vOECTs used vias to connect the S/D contacts that were printed on opposite sides of the substrate (*39*). More recently, micron-scale, photolithographically patterned vOECTs were demonstrated by Donahue *et al*. (*38*), which showed both the scaling behavior and promising cutoff frequency of this OECT device format.

In this work, we target a compact form factor amplifying (voltage-to-voltage) sensing node based on an OECT inverter. We make use of vOECTs in a novel cofacial pair configuration, which has two vOECTs facing one another sharing a single channel. This approach defines an inverter in the same footprint as a single pOECT. The compact configuration does not allow p- and n-type organic mixed conducting ionic-electronic materials (OMIECs) to be patterned separately, necessitating demonstration of an OECT complementary inverter based on an ambipolar OMIEC (*40*). The use of vOECTs allows for the preservation of high gain with reduced area; the cofacial OECT inverter demonstrated a peak gain of about 28. To validate the cofacial OECT inverter as a voltage amplifier, we recorded electrocardiograms (ECGs) from healthy participants with a gain of 10. We further lay out potential barriers to implementation due to degradation and associated p/n imbalance. Last, to demonstrate the robustness and the versatility of the cofacial inverter, we show that it is compatible with a fabrication scheme that makes use of a self-aligning insulation layer and a laser-cut channel, which opens future routes for simplified fabrication, rapid prototyping, and personalized active sensing concepts (*25*).

## RESULTS

### Cofacial OECT pair fabrication and characterization

**Fig. 1.**
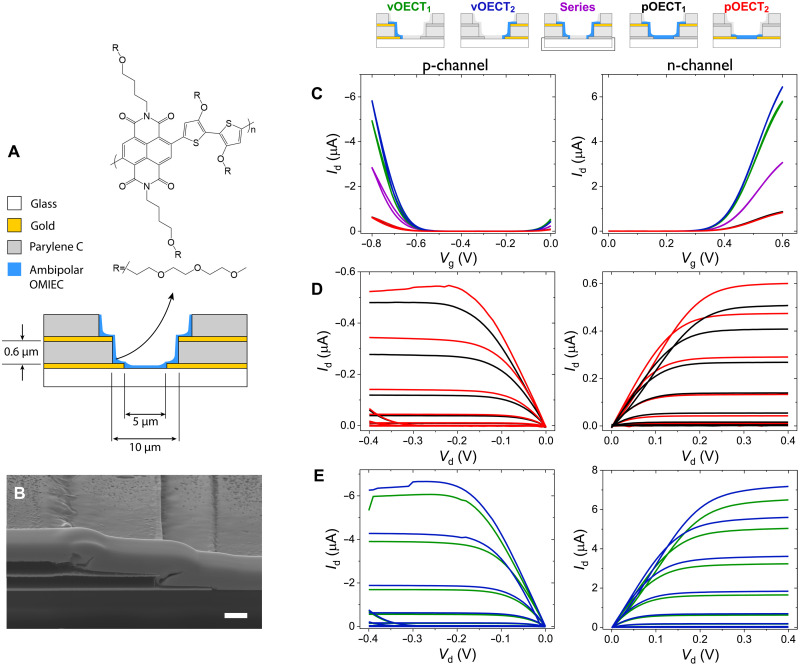
vOECTs and pOECTs in a cofacial pair configuration. (**A**) Cross-sectional schematic of the cofacial pair showing materials, dimensions, and contacts for individual OECT wiring, as well as for the on-chip inverter. Shown above is the chemical structure of p(C_4_-T2-C_0_-EG). (**B**) A tilted scanning electron microscopy (SEM) micrograph of the cross section of the cofacial pair of OECTs. Focused ion beam (FIB) milling was used to expose the cross section, necessitating a thick layer of Pt deposited on top of the completed device. Scale bar, 1 μm. (**C**) Transfer curves of all the possible p-type (Δ*V*_g_ = 0.01 V, 0 to −0.8 V, and *V*_d_ = −0.4 V) and n-type (Δ*V*_g_ = 0.01 V, 0 to 0.6 V, and *V*_d_ = 0.4 V) OECTs (two pOECTs, two vOECTs, and the two vOECTs in series) of the cofacial pair. (**D**) Output curves of the p-type (Δ*V*_g_ = 0.05 V, 0 to −0.8 V, and *V*_d_ = 0 to −0.4 V) and n-type (Δ*V*_g_ = 0.05 V, 0 to 0.6 V, and *V*_d_ = 0 to 0.4 V) bottom and top pOECTs of the cofacial pair. (**E**) Output curves of the p-type (Δ*V*_g_ = 0.05 V, 0 to −0.8 V, and *V*_d_ = 0 to −0.4 V) and n-type (Δ*V*_g_ = 0.05 V, 0 to 0.6 V, and *V*_d_ = 0 to 0.4 V) left and right vOECTs of the cofacial pair.

To achieve a small footprint for the active sensing node, we take advantage of the vertical form factor of vOECTs. By simultaneously patterning two vOECTs along opposite side walls of a single active area, we form a cofacial pair of OECTs that will later form the basis for a complementary inverter (Fig. 1, A and B). These structures are fabricated photolithographically, whereby two metallic layers separated by an insulating layer of parylene C (PaC) serve as the S/D contacts and interconnects. In this case, the thickness of the separating PaC layer roughly defines the vOECT channel length (~600 nm), whereas the width of the OECTs and the spacing between the cofacial vOECT pair are defined photolithographically by the etched area, as noted in Fig. 1A. The on-chip inverter structure requires the bottom contacts to be shorted such that the two vOECTs are in series—this shorted terminal serves as the output signal for the inverter, as discussed below. Full fabrication details can be found in Methods.

To take advantage of the cofacial pair format, we compared the individual operation of the vertical devices (vOECTs) to traditional microfabricated pOECTs. In this work, an OMIEC with both hole and electron transport capability under electrochemical modulation in aqueous electrolyte is desired. The donor-acceptor polymer p(C_4_-T2-C_0_-EG), featuring a naphthalenetetracarboxylic diimide unit with hybrid alkyl-glycol side chains and a triethylene glycol–substituted bithiophene unit, has recently demonstrated ambipolar charge transport in OECTs and therefore meets the above requirements (*40*). The characteristics of this material were validated in a traditional pOECT configuration by analyzing current-voltage (*I-V*) characteristics and electrochemical impedance data. Traditional single-layer planar 100/10-μm (*W*/*L*) pOECTs with 50-nm active layer thicknesses were tested to extract the parameters of p(C_4_-T2-C_0_-EG) (fig. S1). The summary of parameters shown in table S1, which matches those previously reported (*40*), shows that while the transconductance (*g*_m_), mobility (μ_e_) (extracted from *I*_d_-*V*_g_, transfer characteristics), and volumetric capacitance (C^*^) [from electrochemical impedance spectroscopy (EIS)] are similar when operated as hole and electron (p- and n-type) OECTs, the threshold voltages (*V*_th_) are not.

To test the operation of the vOECTs, we chose the top and bottom contacts as the S/D, respectively, while the gate was a Ag/AgCl electrode immersed in a 0.1 M NaCl electrolyte. The resulting transfer and output *I-V* characteristics can be seen in Fig. 1 (C to E). Choosing top right and top left contacts as the S/D terminals is a measure of the two vOECTs in series. Hence, separate photolithographically fabricated test structures with the bottom contact not shorted were used to test both planar device configurations (Fig. 1, C and D). The channel length of the pOECTs is defined as the total OMIEC channel distance between the two contacts. The top pOECT of the pair has a length of 6.2 μm, while the bottom pOECT has a length of 5 μm (Fig. 1). Both the vOECTs and the pOECTs have a channel width of 100 μm and a channel thickness of 150 nm (this thickness is measured at the bottom of the photolithographically defined region, as an accurate determination of film thickness on the side walls is limited). As seen by the output and transfer curves in Fig. 1 (C to E), the drain current of the vOECTs is ~10 times higher than that of the pOECTs, for both p- and n-type, which is on par with the geometric scaling of the OECT channels, assuming comparable device thickness (i.e., current scaling roughly matches channel length scaling). To confirm the series measurement of the vOECTs with the on-chip shorted output, we separately shorted two isolated vOECTs together in series to confirm that the peak current is approximately half the maximum current in the two vOECTs (fig. S2), as expected. The transfer characteristics of the vOECTs of a cofacial pair with the bottom contacts not shorted (fig. S2) is comparable to the vOECTs with the shorted bottom contacts (Fig. 1C).

### Cofacial ambipolar complementary inverter

**Fig. 2.**
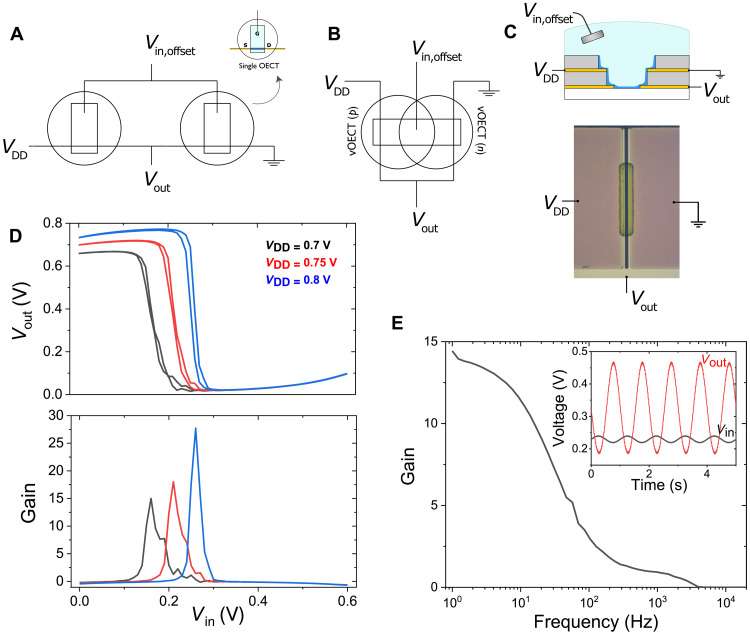
OECT cofacial pair complementary inverter. (**A**) The wiring diagram for an OECT-based complementary inverter with two OECTs independently gated. (**B**) The condensed wiring diagram of an OECT complementary inverter based on a cofacial pair of vOECTs. (**C**) Schematic cross section of a cofacial pair wired as a complementary inverter and a top view microscopic image of the cofacial inverter. (**D**) The voltage transfer characteristics (VTCs) of the cofacial pair inverter (Δ*V*_in_ = 0.01 V; 0 to 0.6 V; and *V*_DD_ = 0.7, 0.75, and 0.8 V). The corresponding gain (δ*V*_out_/δ*V*_in_) with peak gains of 15, 18, and 28 for *V*_DD =_ 0.7, 0.75, and 0.8 V, respectively. (**E**) The frequency response of the cofacial pair. The inset shows a sinusoidal input (Δ*V*_in_ = 0.01 V with an offset of 0.23 V at 1.5 Hz and *V*_DD_ = 0.8 V) with the corresponding amplified output.

A traditional complementary inverter is typically made by separately wiring two different transistors, p- and n-type transistors (Fig. 2A) (*36*, *37*). The source of the p-type OECT is connected to *V*_DD_, while the source of the n-type OECT is wired to ground. The gates of the two OECTs are wired together externally to create a common input. Then, the drain terminals of both OECTs are wired together externally to read *V*_out_.

To achieve such an inverter within the footprint equivalent to that of a single pOECT, we use the cofacial pair of vOECTs as described above. The use of an ambipolar material such as p(C_4_-T2-C_0_-EG) is critical in this case, as patterning opposing side walls to separately define p- and n-type material is not possible. In the cofacial configuration, the shared electrolyte and Ag/AgCl gate contacts both channels, which couples the input of the two OECTs. A proposed revised circuit diagram that better reflects the arrangement of the cofacial pair is shown in Fig. 2B.

With an ambipolar material, one vOECT can behave as the p-type OECT and the other as the n-type OECT, depending on the effective *V*_g_ at each channel. The source terminal of the p-type OECT is connected to *V*_DD_, and the source terminal of the n-type OECT is connected to ground, which are the top contacts of the vOECTs. The shorted bottom contacts of each OECT behave as the drain terminals where the output of the inverter (*V*_out_) is recorded (Fig. 2C). The voltage transfer characteristics (VTCs) of the cofacial inverter pair was recorded with varying *V*_DD_ (0.7, 0.75, and 0.8) (Fig. 2D). The gain of the inverter was extracted from the VTCs (δ*V*_out_/δ*V*_in_) and increased as *V*_DD_ increased, reaching a peak gain of about 28 at *V*_DD_ = 0.8 V (Fig. 2D). At *V*_in_ = 0.26 V with *V*_DD_ = 0.8 V and *I*_DD_ = 72 nA, resulting in a power consumption of about 57.7 nW. The inverter characteristics indicate that the inverter is not balanced: The peak gain at *V*_DD_ = 0.8 V is at *V*_in_ = 0.26 V rather than *V*_in_ = *V*_DD_/2 = 0.4 V. Since the cofacial configuration requires that channel dimensions be equivalent, this mismatch is due to the inherent imbalance in the p- and n-type transports and charging of p(C_4_ -T2-C_0_-EG). Since μ_e_ and C* are similar for both the p- and n-type swings (table S1), the difference in *V*_th_ (δ|*V*_th_| ~ 0.3 V) was determined to be the root cause in the shift in the switching voltage of the cofacial inverter from the ideal *V*_DD_/2. The transfer characteristics of the individual vOECTs of this cofacial pair are shown in fig. S3. Because of the large difference in the *V*_th_ of the p- and n-type swings of p(C_4_-T_2_-C_0_-EG) used in this cofacial configuration, the two vOECTs are not in saturation during the transition region of the VTC as is typical of complementary inverters. When *V*_in_ is low enough for the n-type vOECT of the pair to enter saturation, the p-type vOECT has already left saturation, meaning that operation in linear or subthreshold regime is likely. Across an array of five cofacial pairs, the gain varies from 17 to 25 at a *V*_in_ of 0.23 to 0.26 V (fig. S4).

The AC characteristics of the inverter show a cutoff frequency of about 16 Hz (Fig. 2E). The inset shows a sinusoidal input of 10 mV with an offset of 0.23 V at a frequency of 1.5 Hz, resulting in a gain of about 13 observed at the output for this frequency. The frequency response of the individual p- and n-type vOECTs of the cofacial pair compared to the frequency response of the cofacial inverter swept from low to high and high to low frequencies can be seen in fig. S5. On the basis of the directional sweeps, the frequency response of the p-type vOECT at low frequencies is indicative of degradation, complicating analysis. Accounting for this degradation, it appears that both the p- and n-type vOECTs show comparable cutoffs, which is in agreement with the finding that their C* values are similar (table S1). The frequency response of the inverter, lower than the individual vOECTs, may thus be limited by this degradation and/or other known effects, limiting digital inverter switching speed in the transition region.

Next, the stability of the inverter was explored by applying the input bias where peak gain occurs, *V*_in_ = 0.15 V, and monitoring the change in *V*_out_ over time with a *V*_DD_ = 0.8 V (fig. S6). After holding the inverter at *V*_in_ = 0.15 V for about 13 min, the recorded gain of ~10 before the time study has dropped to ~5 after at that bias. The peak gain of the inverter had shifted to *V*_in_ = 0.12 V and had dropped to 8. In addition, the input of the inverter was pulsed from 0 to 0.6 V with a *V*_DD_ = 0.7 V (fig. S7). The *V*_high_ and the *V*_low_ of the inverter during pulsing decrease and increase, respectively. The transfer curves of the individual vOECTs of the pair and the VTC of the inverter were collected before and after pulsing. Before pulsing, the gain of the inverter was around 13 at a *V*_in_ of 0.14 V; however, after pulsing, the gain at the same *V*_in_ had dropped to 7.5. After pulsing, the peak gain shifted from 0.14 to 0.12 V and had dropped slightly to 12.5. We believe that the imbalance in the decay of the μC* and, thus, drain currents of both the n- and p-type vOECTs contribute to the observed shift and decrease in peak gain.

### Cofacial inverter pair as a voltage amplifier

**Fig. 3.**
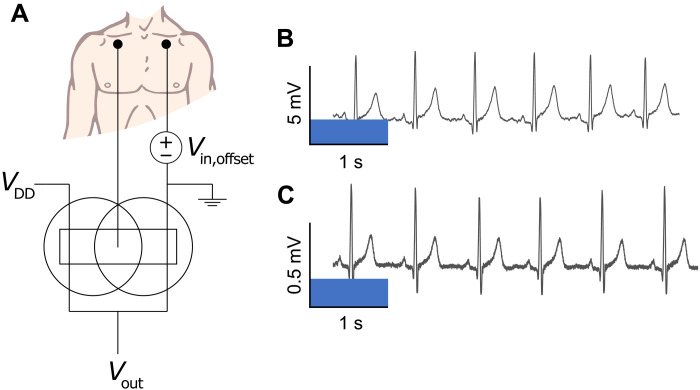
ECG signal amplification using cofacial pair complementary inverter. (**A**) The wiring diagram of the cofacial pair inverter when used as a voltage preamplifier. 3M adhesive medical electrodes are placed below the clavicle on both the right and left side, with one being connected to a DC offset and the other connected directly to the input of the inverter on a benchtop. (**B**) The ECG signal recorded from the output of the cofacial pair inverter. (**C**) The ECG signal recorded directly between the adhesive medical electrodes using a benchtop DMM.

To demonstrate the utility of the cofacial complementary inverter pair for amplifying biosignals, we used the concept as a benchtop preamplifier to record ECG signals. One adhesive medical electrode was connected to the Ag/AgCl gate (input) of inverter, and the other was connected to a voltage supply set to an offset bias where peak gain occurs, 0.26 V, for this cofacial inverter (Fig. 3A). The output ECG had a peak-to-peak amplitude of around 5 mV (Fig. 3B). The potential difference between the two medical electrodes was measured for comparison, which resulted in a peak-to-peak amplitude around 0.5 mV (Fig. 3C). This measured gain of ~10 is consistent with the AC measurements noted above. Other differences in the ECG traces such as noise levels might be attributed to the low cutoff frequency of the inverter as compared to the direct voltage measurement using a digital multimeter (DMM).

### Self-aligned laser-cut cofacial inverter

**Fig. 4.**
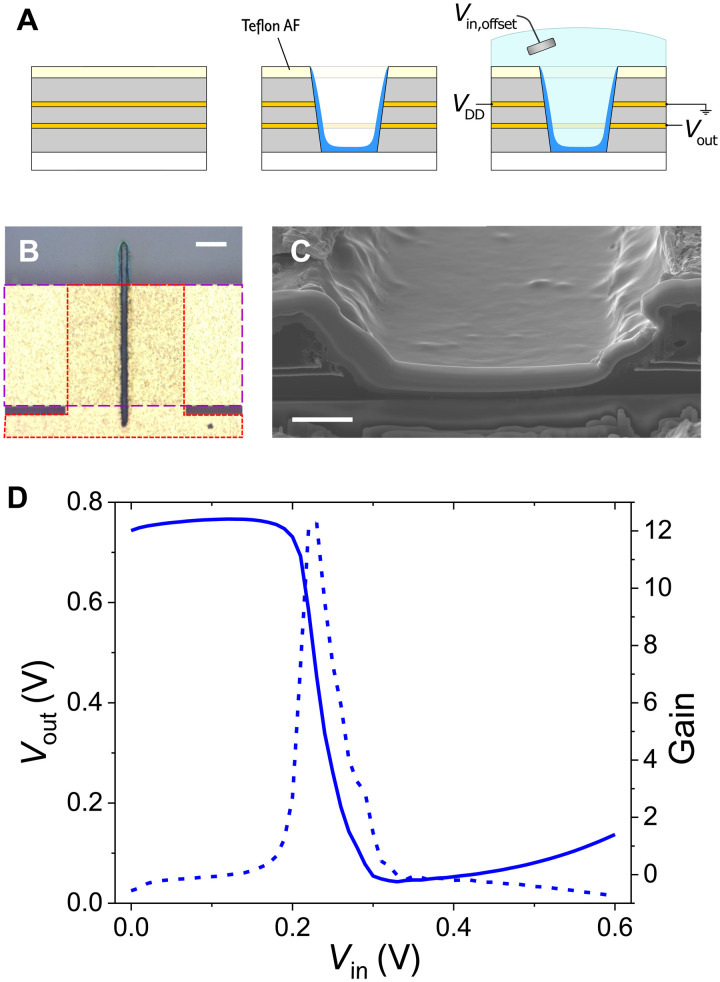
Laser-cut and self-aligned cofacial complementary inverter pair. (**A**) The cross-sectional schematic of a laser cut cofacial pair. The stack has an added hydrophobic coating to aid in the selective deposition of p(C_4_-T2-C_0_-EG) in the laser-cut trench. (**B**) Optical micrograph of the self-aligned laser-cut cofacial pair. The dashed red outline denotes the shorted bottom contacts of the vOECTs where *V*_out_ of the inverter is recorded. The dashed purple box outlines the top contacts of the vOECTs of the inverter that are connected to *V*_DD_ and ground. Scale bar, 200 μm. (**C**) A tilted SEM micrograph of the cross section of the cofacial pair of OECTs. FIB milling was used to expose the cross section, requiring deposition of a thick Pt layer. Scale bar, 5 μm. (**D**) The VTCs of the cofacial pair inverter (Δ*V*_in_ = 0.01 V, 0 to 0.6 V, and *V*_DD_ = 0.8 V) and the corresponding gain (δ*V*_out_/δ*V*_in_) with a peak gain of about 12.

The use of photolithography, above, is a helpful tool to show that the concept of the cofacial pair is viable and promising; however, the fabrication process introduces nonidealities such as large contact overlaps and is not amenable to rapid prototyping and/or personalization. Hence, we show that the cofacial structure is versatile and amenable to a self-aligned laser ablation–based fabrication scheme (*25*). The cofacial inverter pair was assembled using a fabrication scheme previously developed for pOECTs using a laser-cut channel and self-aligning Teflon coating (Fig. 4A). While photolithography was used to pattern the gold contacts, this fabrication method is compatible with printing techniques to eliminate photolithography completely. This fabrication method opens possibilities for simplification of the fabrication of large area electronics and flexible devices for bioelectronics and ubiquitous computing. This self-aligning method overcomes the limit in channel alignment during photolithography to eliminate the channel overlap, which could be beneficial for reducing parasitic capacitance and moving toward higher frequency recording in analog circuits and fast state switching in digital circuits (*4*, *20*, *32*, *38*).

The multilayer layer stack (substrate, two metallic layers, insulating interlayer, and top insulation) is ablated using a laser cutter to create a trench that can be used as the OECT channel. The difference in surface energy between the hydrophobic Teflon coating and the hydrophilic laser-cut channel allows the p(C_4_-T2-C_0_-EG) to wick into the channel and the remaining material to be dragged away. The optimization of laser power to ablate the stack can be seen in fig. S8. A laser power of 2 W was chosen to ensure that the gold and PaC layers were fully ablated; at lower power, we can see gold residue in the channel and PaC surrounding the channel, but these cutting conditions are not sufficient to damage the glass carrier substrate. The current incarnation of this method uses a picosecond laser, which leads to significant local heating and thus ample damage, as is evident in Fig. 4, B and C. While the devices do not provide the clean cuts of a vertical side wall as depicted in the schematic, they yield operational cofacial pair complementary inverters (Fig. 4D), which operate under the same principles as those in Fig. 2. In the case of the self-aligned, laser-cut devices, the exact and uniform dimensions of the separate devices are not readily discernable due to the ill-defined device topography and the variations in active material thickness owing (effectively) to a drop-casting method. These same factors likely contribute to the difference in performance between the self-aligned laser-cut and photolithographically defined inverters. The damaged laser-cut sidewalls of the channel might change the effective length of the vOECT channels and affect material contact with the S/D contacts, as opposed to the photolithographically defined channels, which have smoother vertical sidewalls (Fig. 1B).

## DISCUSSION

The concept of a cofacial arrangement of OECTs presented here enables an ambipolar inverter where the composite OECTs are colocalized in a manner that allows for a compact geometry. When implemented with vOECTs, the inverter active area (input) spans the same footprint as a single pOECT. vOECTs not only offer compact geometry but also, when compared to pOECTs of the same area and thickness, offer a 10-fold increase in the drain current (Fig. 1). Geometric dimensions affect the gain of the inverter, as the gain is directly proportional to *g*_m_ (*37*). Shorter length channels can result in a higher gain, and while the thickness of the channel can be increased to compensate for longer channel lengths, it increases the response time, making it less suitable for electrophysiological recordings (*37*). It is difficult to directly compare the inverter of the cofacial pair and an inverter based on two pOECTs wired together externally due to the challenges in measuring and controlling the film thickness of the vOECT channels. However, an inverter with pOECTs with the same area (10 μm by 100 μm) as the cofacial inverter pair with the channel material, p(C_4_-T2-C_0_-EG), shows a peak gain of 8 (fig. S1) compared to 28 for the cofacial vertical configuration (Fig. 2).

The ambipolar inverters demonstrated here using p(C_4_-T2-C_0_-EG) are not perfectly balanced (n versus p channel), which can be problematic for digital logic applications. In this cofacial configuration, it would not be feasible to independently change the dimensions (i.e., length and thickness) of the individual p- and n-type vOECTs to achieve a balanced inverter; however, the resulting VTCs may be beneficial in analog applications. Since *V*_th_ of the p-type OECT is higher than the *V*_th_ of the n-type OECT, the input voltage at which peak gain occurs is shifted closer to *V*_in_ = 0 V, which means that the offset required when recording biological signals is smaller, reducing power consumption. In addition, the use of an ambipolar material may present some difficulties for digital logic circuits, as the inverter does not fully pull up to *V*_DD_ or pull down to ground. This is due to the p- and n-type OECTs not fully turning off, as seen from the transfer curves of the vOECTs (fig. S3), as they enter an ambipolar regime of operation (*41*–*45*). This feature in the VTC has also been observed in previously reported organic field-effect transistor–based ambipolar inverters (*44*–*46*). However, when used as a voltage amplifier, the nonidealities of an ambipolar inverter do not adversely affect its performance.

The developed cofacial OECT complementary inverter is used as a proof-of-concept on-board voltage amplifier to record electrophysiological signals. This single inverter can used to replace traditionally used voltage dividers to reduce the overall footprint of the active sensing node. While the cutoff frequency of the inverter is high enough to record low-frequency biological signals such as ECG, the geometry can be further modified to reduce response time to ensure no attenuation of higher frequency activity. This includes shorter channel widths and decreasing the thickness of the insulating layer between the S/D contacts of the vOECTs by reducing PaC thickness or targeting thinner conformal oxide layers to achieve shorter vOECT channel length. This approach may add challenges in fabrication and reproducibility and increase contribution of parasitic lead resistance and/or short channel effects (*38*). Another route to achieve higher frequency cutoff is to decrease the channel thickness, which, however, is likely to decrease the gain of the inverter (*37*). As with single OECT sensors, it appears that a balance must be navigated between gain and response time. Another consideration when using an inverter is the allowable input window when operating about the bias where peak gain occurs. When gain increases the linear, window of operation decreases so the amplitude of the biological signal of interest must be considered when designing the inverter; a balance between high gain and minimal distortion should be achieved. On the basis of the VTC of the cofacial inverter presented here (Fig. 2D), the maximum input signal amplitude resulting in minimal output distortion is around 40 mV, which allows for the recording of many biological signals that fall in the microvolt to millivolt range such as the ECG, electroencephalography, and even electrocorticography recordings (*19*, *27*).

The self-aligned, laser-cut cofacial inverter shows that this device concept is compatible with direct write fabrication techniques, which is enabled by the relaxed fabrication constraints of OECTs owing to their bulk transport properties. This fabrication method can address the issue of patterning p- and n-type materials in separate channels when an ambipolar material is not available owing to the Teflon AF topcoat. This feature may allow for separate materials to be dragged and dropped into adjacent perforations of the materials stack. Separate p- and n-type materials may be desirable for digital circuits to ensure the complete switching to the logic level high and low but not necessary for analog applications. Thus, self-aligning vOECTs for complementary logic gates can be integrated into more complex logic circuits such as decoders, which are typically composed of hundreds of OECTs and resistors to make up unipolar gates, to enhance performance and potentially reduce the overall footprint. Despite the potential advantages presented by the laser-cut patterning approach, further development and optimization of the process are needed. To improve the performance and reproducibility of the self-aligned OECTs, different ablation methods such as femtosecond laser cutting and focused ion beam (FIB) milling can be used. Excessive local heating and associated damage must be minimized, for example, using shorter duration pulses and exploring processing and postprocessing conditions for ablation of the particular materials stack to enhance the topography of the channels.

Last, while the in-sensor signal amplification of this cofacial pair inverter concept presents numerous advantages, significant barriers must be addressed before practical adoption in bioelectronics. First, reproducibility must be improved regardless of fabrication approach. Without inverter-to-inverter reproducibility, an array of these active sensing nodes will require a different *V*_DD_ and/or *V*_in_ offset to achieve the same gain. Second, the stability of the p(C_4_-T2-C_0_-EG) herein prevents this inverter from achieving long-term use, critical for both in vivo and wearable applications. This work suggests that both n- and p-type performance of this particular material degrade on both extended switching and during continuous operation at the *V*_in_ of peak gain. Device degradation affects both the individual OECT characteristics, affecting peak gain of the inverter, but more significantly exacerbates loss in gain at the chosen operating point by causing a shift in the switching voltage. The understanding of both of these effects is limited also by the lack of significant ambipolar OMIEC candidates (*40*, *43*). Further work is needed to understand the mechanism of degradation and to improve the stability of ambipolar materials. Existing studies exploring stability, degradation mechanisms, and side reactions in OMIEC materials will inform these efforts (*47*, *48*).

Here, we present an OECT-based ambipolar inverter, which fits within the same area as a typical micron-scale pOECT using a cofacial pair of vOECTs. The compact geometry and the peak gain of 28 of these cofacial ambipolar inverters makes them good candidates for direct voltage-to-voltage amplification. We demonstrate the use of the cofacial inverter as a voltage amplifier by recording ECG signals. The signals recorded from the output of the inverter showed a 10× amplitude when compared to direct voltage recordings using a DMM. We further fabricated the cofacial inverter using self-aligned, laser-cut vOECTs that open new avenues for simplified fabrication and large area printed electronics. While we outline barriers that must be overcome for this device concept, the cofacial inverter concept herein presents a promising approach toward on-site and in-sensor amplification. This colocalization of a simple circuit using vOECTs may be further applicable to other on-site signal processing functions including rectification and reference-based differential biochemical detection on flexible and conformal bioelectronic arrays. This active sensing scheme can therefore be used to achieve functional back-end processing at or near the biotic/abiotic interface for use in far-reaching biointegrated devices.

## METHODS

### Device fabrication

The cofacial inverters were fabricated using a dry peel-off process reported previously but will be mentioned here briefly (*25*, *27*). To define the first layer of gold, AZ nLOF 2035 was spun onto clean microscope slides at 3500 rpm and then baked at 110°C for 1 min. The slides were then exposed to ultraviolet (UV) light using an MJB4 mask aligner, then baked at 110°C for 1 hour and 30 min and developed in AZ 300 MIF for 30 s. The slides were then placed in an AJA E-Beam where 5 nm of Cr and 100 nm of Au were deposited and then left in acetone for liftoff for 30 min. A 0.65-μm-thick PaC [Specialty Coating Systems (SCS) Coatings] insulation layer was then deposited in the presence of the adhesion promoter A 174 using a SCS Labcoter 2. Another gold layer was patterned on top of the insulation layer using the same steps mentioned above. Once the final insulation layer was deposited, an antiadhesive was spun on, and a sacrificial layer of PaC was deposited. To pattern the active sites and contact pads AZ P4620 was spun on at 3000 rpm and baked at 110°C for 2 min. It was then exposed to UV light using an MJB4 mask aligner and developed in 1:4 AZ 400K. The slides were then placed in a Samco reactive ion etcher to selectively etch the PaC using CHF_3_ and O_2._

The polymer p(C_4_-T2-C_0_-EG) was synthesized following a previously reported protocol (*40*). Then, p(C_4_-T2-C_0_-EG) was spun on at 900 rpm from a solution (5 mg/ml), and the sacrificial layer was mechanically peeled off, leaving the material only in the channel. Standard pOECTs were made using the same steps but without a second gold layer. The laser-patterned self-aligned cofacial pairs were made using the same steps, but a sacrificial PaC layer was deposited on the glass slide first, and after the final insulation layer was deposited onto the second layer of gold, a layer of Teflon AF 2400 was spun onto the slide at 1500 rpm and baked at 210°C for 20 min. Then, using an LPKF laser cutter, the channels were cut using a power of 2 W, a frequency of 40 kHz with one repetition. A droplet of p(C_4_-T_2_-C_0_-EG) was then pinned onto the Teflon coating and dragged and dropped into the ablated channel. All thickness measurements were performed using a Veeco Dektak-8 stylus profilometer. Cross-sectional scanning electron microscopy (SEM) images were taken using a JEOL 4700F FIB/SEM. First, 20 nm of bulk platinum (Pt) was sputter-coated, and then 1 μm of local Pt was deposited to prevent ion beam damage. The cross sections were milled using a FIB at 30 kV with currents up to 10 nA. The samples were mounted at 45° and tilted at 20° for SEM imaging at 10 kV. Microscopic images were taken using a Zeiss Scope.A1 and Axiocam 105 color.

### Electrical characterization

OECT and inverter measurements were made with a National Instruments (NI) PXIe 1082 using custom LabVIEW code. In addition, all measurements were performed in 0.1 M NaCl using a Ag/AgCl electrode as an external gate. Output curves and transfer curves were collected using source measuring units (SMUs) (NI PXIe-4143). For the p-type measurements, the output curves were collected using a *V*_d_ from 0 to −0.4 V and a *V*_g_ from 0 to −0.8 V, and transfer curves were collected using a constant *V*_d_ = −0.4 V, while varying *V*_g_ from 0 to −0.8 V. For the n-type measurements, the output curves were collected using a *V*_d_ from 0 to 0.4 V and a *V*_g_ from 0 to 0.6 V, and transfer curves were collected using a constant *V*_d_ = 0.4 V, while varying *V*_g_ from 0 to 0.6 V.

The threshold voltage (*V*_th_) of the p- and n-type OECTs were extracted from a linear extrapolation of the √|*I*_d_| versus *V*_g_ plot. EIS was performed using a potentiostat (Metrohm Autolab) to extract volumetric capacitance (C*). A three-electrode configuration was used with a 10-mV sine wave with offsets from 0.5 to 0.8 V for the p-type direction and −0.2 to −0.5 V for the n-type direction. The spectra were recorded from 0.1 to 10^5^ Hz. A Randall’s circuit [R(R|C)] was fit to the impedance spectra to extract the effective capacitance, which was normalized by film volume to find volumetric capacitance (C*). Mobility of both the holes and electrons was extracted using the following equation $g_{m}=\frac{\mathrm{wd}}{L}\mu C^{*}(V_{g}-V_{\text{th}})$.

The VTCs of the inverter were collected using the SMUs to apply *V*_DD_ and *V*_in_, and a DMM (NI PXIe-4081) was used to measure *V*_out_. The VTCs were collected by sweeping *V*_in_ from 0 to 0.6 V while holding *V*_DD_ constant at 0.7, 0.75, or 0.8 V. Gain was calculated by taking the derivative of the VTC (δ*V*_out_/δ*V*_in_). AC measurements on the inverter were performed by applying an input of 10-mV sinusoidal signal with an offset at the peak gain of the inverter at varying frequencies using the NI PXIe 6363. Stability studies were performed by pulsing *V*_in_ from 0 to 0.6 V in 5-s intervals using the NI PXIe 6363.

ECG was measured by placing two adhesive 3M dot electrodes right below the clavicle on the right side and left side of the participant. One 3M electrode was wired directly to the Ag/AgCl gate of the inverter (on the benchtop), while the other 3M electrode was wired to a bias (NI PXIe 6363). For comparison, the two 3M dot electrodes were wired directly to the DMM. The signals were acquired at 1 kHz and filtered using a bandstop filter of 55 to 65 Hz and then a bandpass filter of 0.1 to 100 Hz. All ECG measurements were taken in compliance with the Institutional Review Board guidelines and with informed written consent before subject participation.
