# Supplementary material for: Ambipolar inverters based on cofacial vertical organic electrochemical transistor pairs for biosignal amplification
Source: Sci Adv. 2021 Sep 8;7(37):eabh1055. doi: 10.1126/sciadv.abh1055 (PMC8442873; doi:10.1126/sciadv.abh1055)
Supplement: Supplementary file 1 — Figs. S1 to S8 Table S1 [file sciadv.abh1055_sm.pdf]

Science Advances
AAAS

Supplementary Materials for

Ambipolar inverters based on cofacial vertical organic electrochemical transistor pairs for biosignal amplification

Reem B. Rashid, Weiyuan Du, Sophie Griggs, Iuliana P. Maria, Iain McCulloch, Jonathan Rivnay*

*Corresponding author. Email: jrivnay@northwestern.edu

Published 8 September 2021, *Sci. Adv.* **7**, eabh1055 (2021)
DOI: 10.1126/sciadv.abh1055

This PDF file includes:

Figs. S1 to S8
Table S1

Table S1: Device characteristics of planar p(C_4-T2-C_0-EG) OECTs. This table summarizes the transconductance (g_m) normalized by geometric dimensions (Wd/L), threshold voltage (V_{th}), volumetric capacitance (C^*), and mobility (μ_e) of the ambipolar material p(C_4-T2-C_0-EG) in both the n- and p-operation. Transistor characteristics were extracted from N=6 devices, errors reported are standard deviations.

	g_m (S/cm)	V_{th} (V)	C^* (F/cm^3)	μ (cm^2/Vs)
p-channel	$2.73 \times 10^{-2} \pm 1.69 \times 10^{-2}$	$-0.60 \pm 9.11 \times 10^{-3}$	90.8 ± 17.03	$1.48 \times 10^{-3} \pm 9.08 \times 10^{-4}$
n-channel	$3.07 \times 10^{-2} \pm 1.82 \times 10^{-2}$	$0.30 \pm 4.76 \times 10^{-2}$	125 ± 34.60	$1.28 \times 10^{-3} \pm 7.74 \times 10^{-4}$

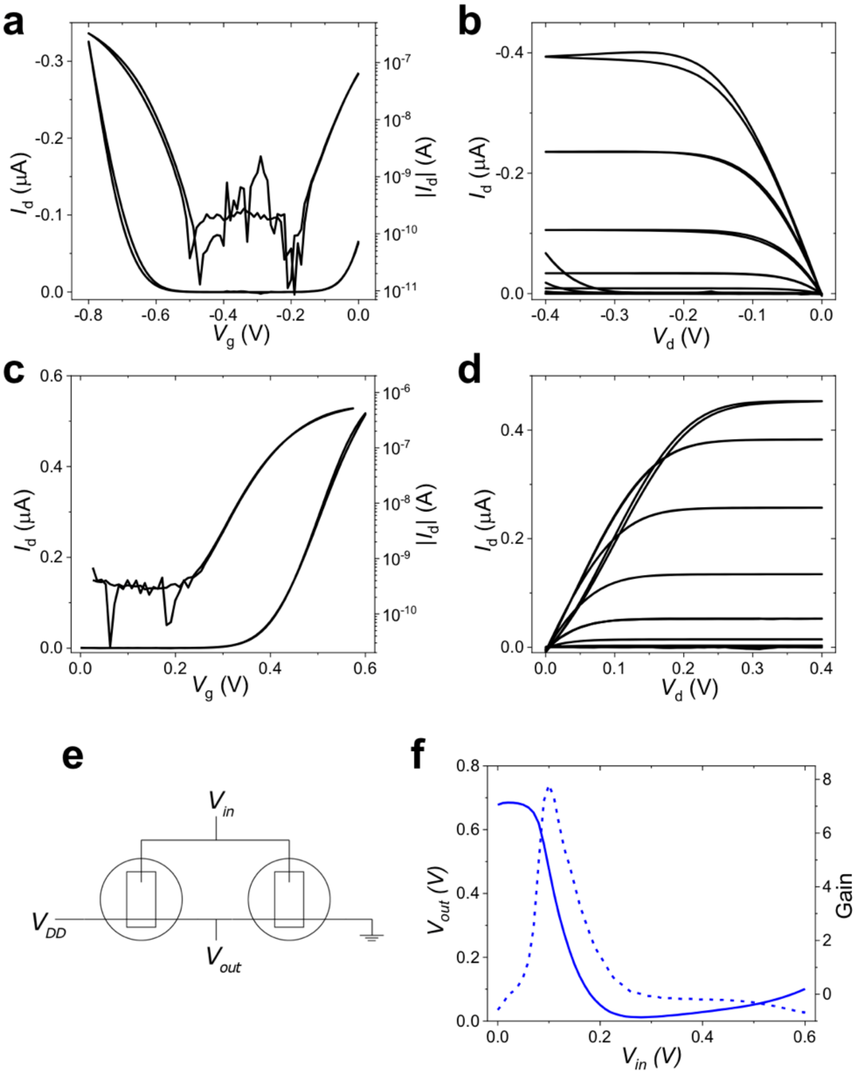

Figure S1: Characterization of planar OECTs. a. Transfer curve (ΔV_g = 0.01 V, 0V to -0.8V and V_d = -0.4V) on a log and linear scale of a p-type pOECT. b. Output curve (ΔV_g = 0.05 V, 0 V to -0.8V and V_d : 0 to -0.4V) of a p-type pOECT. c. Transfer curve (ΔV_g = 0.01 V, 0V to 0.6V and V_d = 0.4V) on a log and linear scale of a n-type pOECT. d. Output curve (ΔV_g = 0.05 V, 0 V to 0.6V and V_d : 0 to 0.4V) of a n-type pOECT. e. The wiring diagram of a complementary OECT-based comprised of two complementary pOECTs externally wired to one another on the same substrate. f. The voltage transfer characteristic of the inverter described in e) (ΔV_g = 0.01 V, 0V to 0.6V and V_{DD} = 0.8V) and the corresponding gain ($\delta V_{out}/\delta V_{in}$) with a peak gain of around 8.

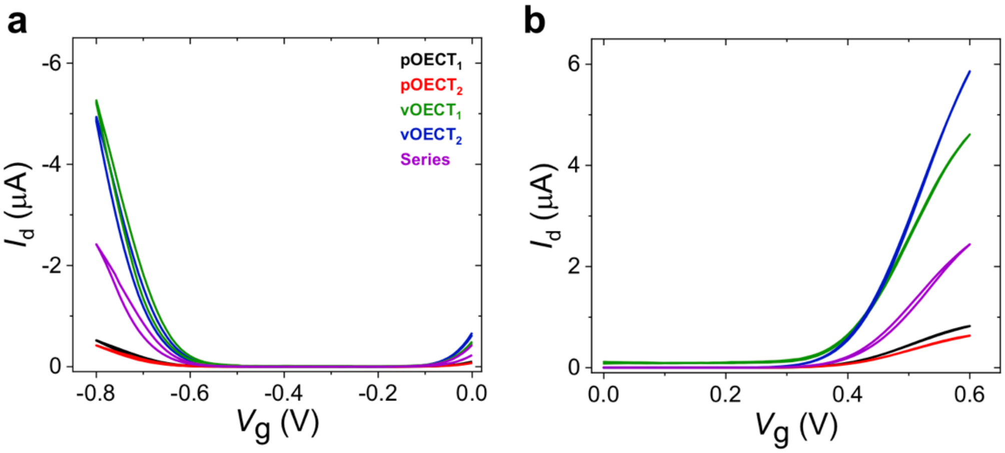

Figure S2: Transfer curves of cofacial pairs. a. P-type transfer curves of all possible OECT combinations (2 pOECTs, 2 vOECTs, and the 2 vOECTs in series) in a cofacial pair with isolated bottom contacts (ΔV_g = 0.01 V, 0V to -0.8V and V_d = -0.4V). b. N-type transfer curves of all possible OECT combinations (2 pOECTs, 2 vOECTs, and the 2 vOECTs in series) in a cofacial pair with isolated bottom contacts (ΔV_g = 0.01 V, 0V to 0.6V and V_d = 0.4V).

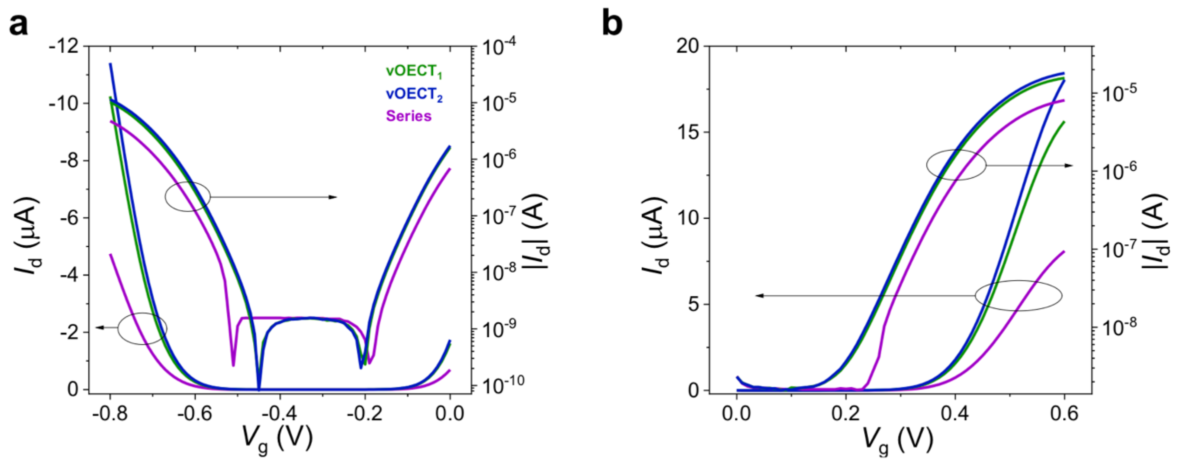

Figure S3: Transfer curves of cofacial inverter pair. a. P-type transfer curves of all possible OECT combinations (2 pOECTs, 2 vOECTs, and the 2 vOECTs in series) in a cofacial pair with shorted bottom contacts (ΔV_g = 0.01 V, 0V to -0.8V and V_d = -0.4V) on a log and linear scale. b. N-type transfer curves of all possible OECT combinations (2 vOECTs and the 2 vOECTs in series) in a cofacial pair with shorted bottom contacts (ΔV_g = 0.01 V, 0V to 0.6V and V_d = 0.4V) on a log and linear scale.

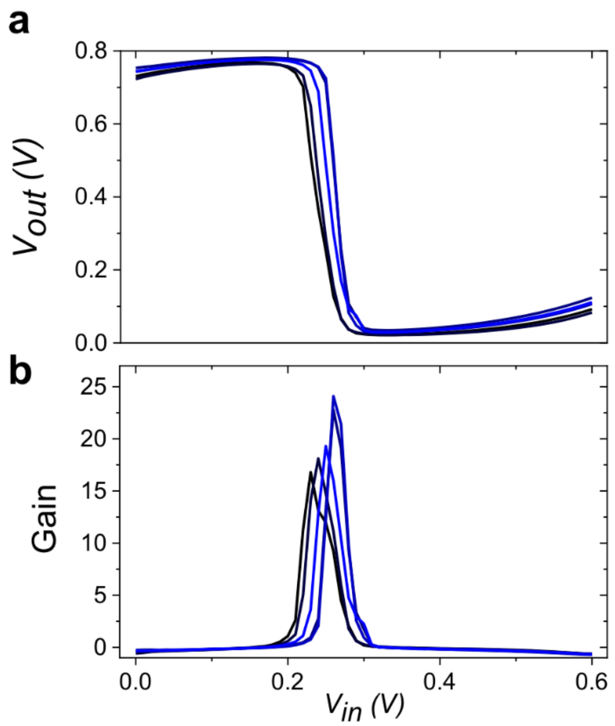

Figure S4: Voltage transfer characteristics of cofacial inverter array. a. The voltage transfer characteristics of an array of 5 cofacial complementary inverters (ΔV_g = 0.01 V, 0V to 0.6V and V_{DD} = 0.8V. b. The corresponding gain ($\delta V_{out}/\delta V_{in}$) with peak gains that vary from 17 to 25.

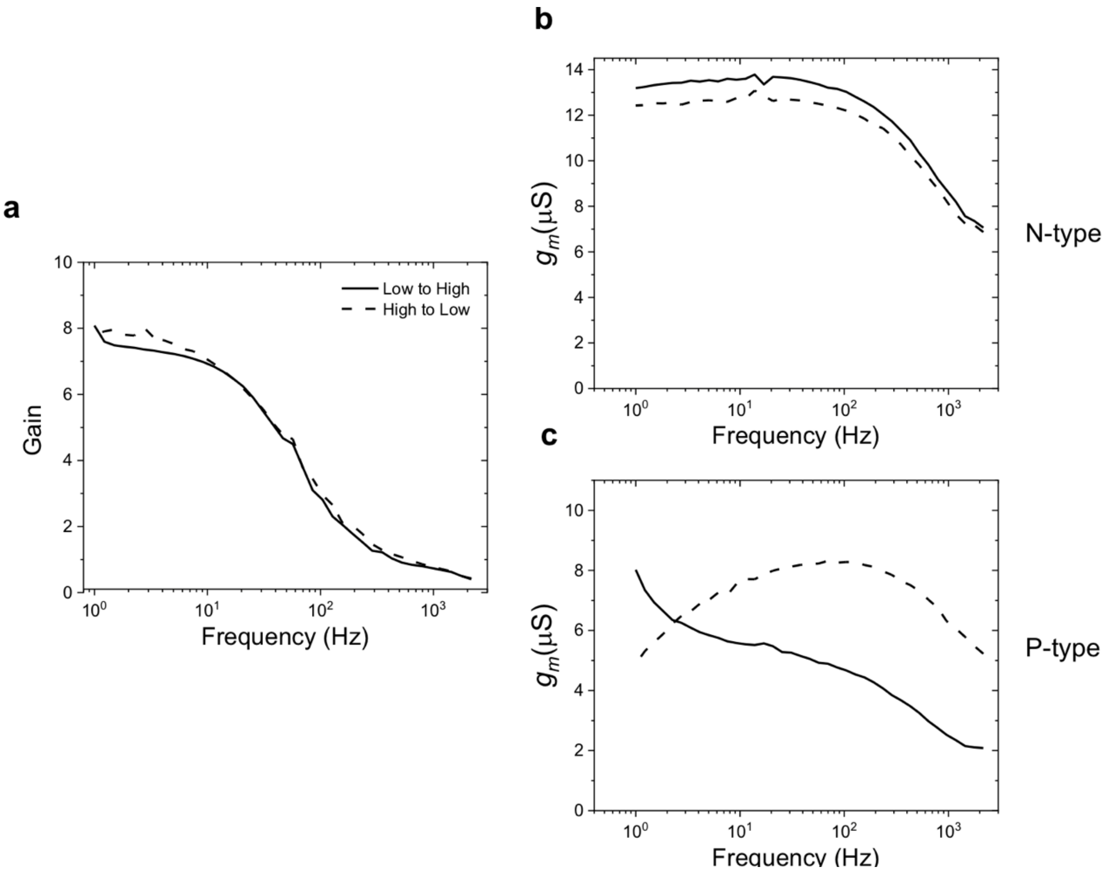

Figure S5: **Frequency response of cofacial inverter pair.** a. The input of the cofacial inverter swept from low to high frequency (solid) and from high to low frequency (dashed) (V_{in} = 0.23 V, Δ0.01 V; V_{DD} = 0.8V). b. The input of the n-type vOECT of the cofacial pair swept from low to high frequency (solid) and from high to low frequency (dashed) (V_g = 0.6 V, Δ0.01 V; V_d = 0.4V). c. The input of the p-type vOECT of the cofacial pair swept from low to high frequency (solid) and from high to low frequency (dashed) (V_g = -0.8 V, Δ0.01 V; V_d = -0.4V). It is evident that the p-type vOECT suffers from degradation, most evident in the low frequency regime.

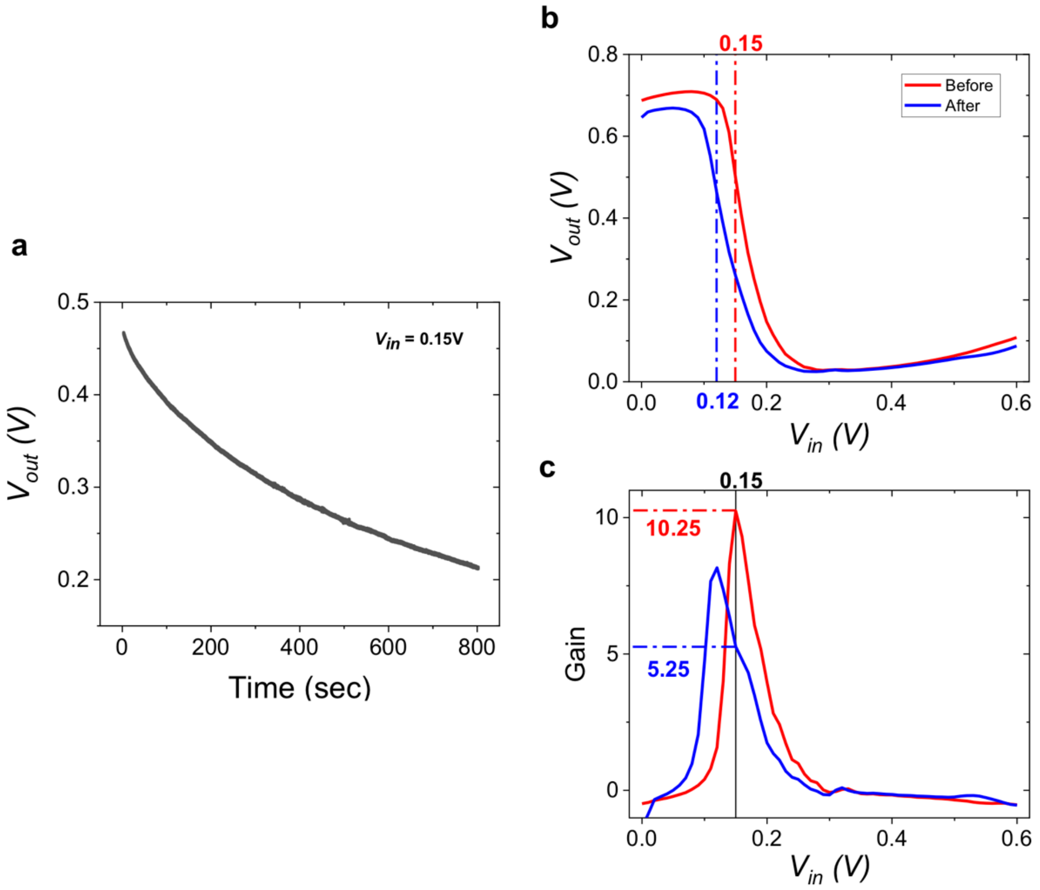

Figure S6: Stability study of cofacial inverter. a. The cofacial inverter was held at a constant V_{in}= 0.15V (V_{DD} = 0.8V) where the peak gain of the inverter is observed. The V_{out} of the inverter starts at 0.45V and decays to 0.21V over the course of 13 minutes. b. The voltage transfer characteristics of the cofacial pair before being held at V_{in}= 0.15V and after (ΔV_g = 0.01 V, 0V to 0.6V and V_{DD} = 0.8V). There is an observed shift in the switching voltage of the VTC; the peak gain has shifted from V_{in}= 0.15V to V_{in}= 0.12V. c. The corresponding gain ($\delta V_{out}/\delta V_{in}$) before and after pulsing with an observed shift of the peak gain and has decreased by about 5.

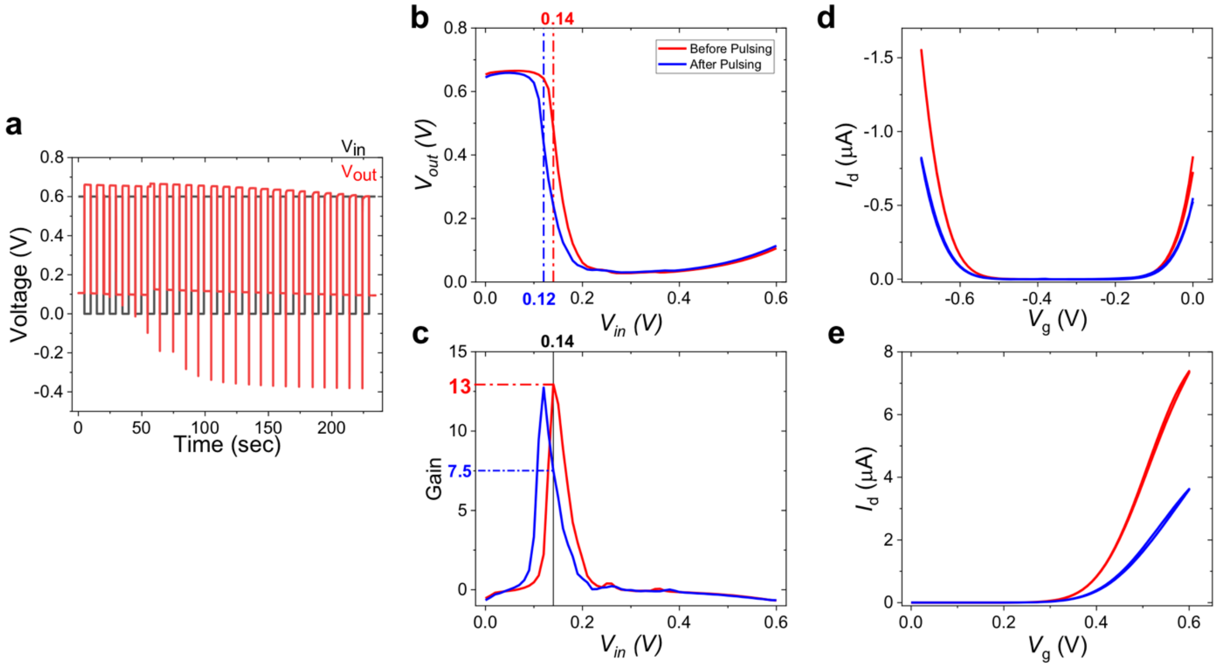

Figure S7: Cofacial inverter's response to pulsing. a. Pulsing the input of the cofacial pair from 0 to 0.6V (V_{DD} = 0.7V) for about 4 minutes and the resulting output. The $V_{out,low}$ increases while the $V_{out,high}$ decreases. b. The voltage transfer characteristics of the complementary inverter before and after pulsing (ΔV_g = 0.01 V, 0V to 0.6V and V_{DD} = 0.7V). There is an observed shift in the switching voltage of the VTC. c. The corresponding gain ($\delta V_{out}/\delta V_{in}$) before and after pulsing with an observed shift of the peak gain. d. P-type transfer curves (ΔV_g = 0.01 V, 0V to -0.7V and V_d = -0.4V) of one of the vOECTs in the cofacial pair before and after pulsing. E) N-type transfer curves (ΔV_g = 0.01 V, 0V to 0.6V and V_d = 0.4V) of one of the vOECTs in the cofacial pair before and after pulsing.

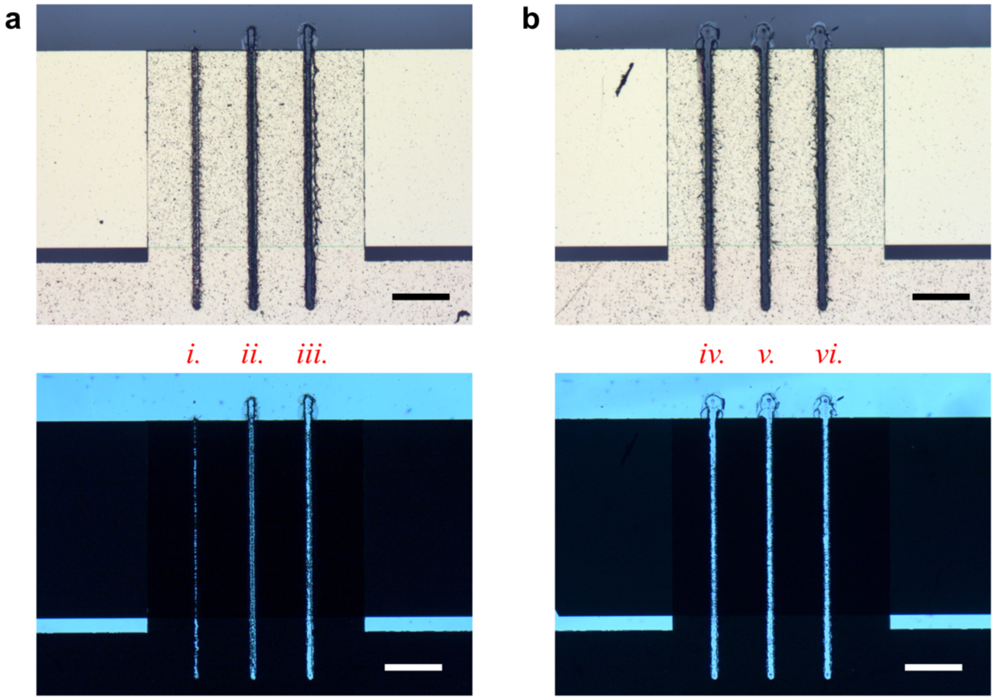

Figure S8: Optimization of laser power for ablation of self-aligned cofacial pair. a. Optical images in reflection (top) and transmission (bottom) mode of the self-aligned cofacial stack cut at laser powers of 0.1W *(i),* 0.5W *(ii),* 1W *(iii).* b. Optical images in reflection (top) and transmission (bottom) mode of the self-aligned cofacial stack cut at laser powers of 1.5W *(iv),* 2.0W *(v),* 2.5W *(vi).* 2W was ultimately selected as the optimal power as it is high enough to cut the PaC but with minimal damage done to the glass substrate. All scale bars are 200µm.

.
